# PI3K/AKT/mTOR Signaling Network in Human Health and Diseases

**DOI:** 10.3390/cells13171500

**Published:** 2024-09-06

**Authors:** Tolulope O. Omolekan, Jean Christopher Chamcheu, Claudia Buerger, Shile Huang

**Affiliations:** 1 Department of Biological Sciences and Chemistry, College of Sciences and Engineering, Southern University and A&M College, Baton Rouge, LA 70813, USA; tolulope.omolekan@bowen.edu.ng; 2 Department of Pathological Sciences, School of Veterinary Medicine, Louisiana State University, Baton Rouge, LA 70803, USA; 3 Department of Biochemistry, Bowen University, Iwo 232101, Nigeria; 4 Department of Pathology and Translational Pathobiology, Louisiana State University Health Sciences Center, Shreveport, LA 71103, USA; 5 Department of Dermatology, University Hospital Frankfurt, Goethe University Frankfurt, Theodor-Stern-Kai 7, 60590 Frankfurt am Main, Germany; 6 Department of Biochemistry and Molecular Biology, Louisiana State University Health Sciences Center, 1501 Kings Highway, Shreveport, LA 71103, USA; 7 Department of Hematology and Oncology, Louisiana State University Health Sciences Center, 1501 Kings Highway, Shreveport, LA 71103, USA; 8 Feist-Weiller Cancer Center, Louisiana State University Health Sciences Center, 1501 Kings Highway, Shreveport, LA 71103, USA

## 1. Introduction

Transduction of molecular signaling is a fundamental mechanism that allows a living cell to communicate internally with other cells and its environment through chemical or physical signals, thereby maintaining its structural integrity and triggering physiological responses. The human body, a complex multicellular entity, has an extensive and coordinated network of signaling pathways necessary for its health, survival, and functionality. These pathways enable the body to maintain homeostasis in response to a range of internal and external stimuli, both under normal and disease conditions, throughout different stages of life. Dysregulation of cell signaling is associated with the onset of many diseases, including cancer, infections, chronic inflammation, as well as neurological, developmental, metabolic, and cardiovascular disorders. Such dysregulations arise from a variety of factors, such as alterations of genes, transcription factors, splicing and chromatin regulators, and abnormal levels of signaling molecules, leading to disruption of the regulatory networks essential for cell function and communication [1]. Since Claude Bernard first introduced the concept of signaling in 1855, research into the molecular complexities of cell signaling in health and disease has spurred the discovery of disease biomarkers, new drug targets, and the development of innovative therapeutic strategies. The PI3K/AKT/mTOR pathway, a highly conserved intracellular pathway in eukaryotic cells, plays a vital role in cell metabolism and regulates various cellular events such as cell growth, proliferation, survival, motility, adhesion, and differentiation [2]. Frequent dysregulation of this pathway in numerous diseases has made it a focus of research to identify biomarkers and define therapeutic targets associated with this signaling cascade. The phosphoinositide 3-kinase (PI3K) can be activated by receptor tyrosine kinases (RTKs), such as the platelet-derived growth factor receptor (PDGFR) or epidermal growth factor receptor (EGFR), which promote cell proliferation and migration, the insulin-like growth factor receptor (IGFR), which stimulates cell growth and survival, and the insulin receptor (IR), which maintains metabolic homeostasis. To synchronize the various cellular responses to multiple external stimuli, PI3K effectors modify multiple physiological aspects of the cell. For example, signals that propel cell cycle progression are synchronized with those that increase the need for metabolic pathways to generate the necessary energy and macromolecules for cell growth and division. PI3K mediates the synthesis of phosphatidylinositol (3,4,5)-trisphosphate (PIP3) in the cells, which acts as a lipid second messenger and recruits AKT (also known as protein kinase B (PKB)) and phosphoinositol-dependent kinase (PDK1) to the membrane. PDK1 can then activate AKT by phosphorylation at Thr308. Complete activation of AKT also requires phosphorylation at Ser473 by mTOR complex 2 (mTORC2) [3]. Fully activated AKT then phosphorylates a variety of signaling molecules with diverse functions and coordinates a complex set of metabolic responses to meet these needs while maintaining homeostasis. Through a feedback loop, the network is regulated, preventing abnormal cell proliferation under nutrient scarcity and other stress conditions. The mTOR complex 1 (mTORC1) phosphorylates and stabilizes the growth factor receptor-bound protein 10 (GRB10), an adaptor protein that binds and inhibits IGFR and IR [4]. Mutations in *GRB10*, *PIK3CA* genes, and other genes in the PI3K-AKT-mTOR pathway result in complex pathologies in humans like type 2 diabetes, congenital lipomatous overgrowth, vascular malformations, epidermal nevis, spinal/skeletal anomalies/scoliosis (CLOVES) syndrome, immune-mediated inflammatory conditions, and hyperproliferative disorders [5]. Understanding the molecular and physiological intricacies of the PI3K/AKT/mTOR signaling network could facilitate the development of new therapies that target this network in pathological conditions without disrupting normal tissues. Such drugs specifically target mutant forms of oncogenic proteins (e.g., p110α with mutant H1047R), thus sparing endogenous signaling molecules, which are critical for the maintenance of normal homeostasis.

Therapeutic strategies targeting this signaling network have been extensively explored, especially in conditions like cancer, neurodegenerative disorders, inflammation, autoimmune diseases, obesity, and diabetes. These strategies include the use of nutraceuticals, synthetic small-molecule inhibitors, and combinations with drugs to enhance efficacy. Preclinical and clinical studies are ongoing to develop these targeted therapies [6]. The U.S. Food and Drug Administration has approved certain inhibitors of PI3K, AKT, and mTOR for some types of cancer, including breast cancer with specific genetic mutations and chronic lymphocytic leukemia [7]. However, it is important to note that while some drugs have been approved, others are still under evaluation or have not received approval for the treatment of any human disease. 

This Special Issue comprises published original research and high-quality reviews of scientific literature that deepen our understanding of the PI3K/AKT/mTOR pathway and its biology. In particular, it encompasses investigations into the roles of this pathway in various human diseases (e.g., cancer, psoriasis, viral infection, and myocardial infarction) and treatments, as well as the delivery of programmed phospho-variants of AKT1 into cells. The findings not only underscore its involvement in various disease conditions but also examine the diverse effects of hotspot mutations in the *PIK3CA* gene on cancer progression. Furthermore, it highlights novel technologies for selectively studying AKT1 biology and the mechanisms of action of different treatment modalities.

## 2. An Overview of Published Articles

Siddika et al., Ghodsinia et al., Ferreri et al., and George et al. independently reported the biology, activity, genome-wide transcriptomic changes, and the consequences of mutations of important effectors of the signaling network in disease conditions. Siddika et al. (contribution 1) described the development of a novel and effective delivery system for programmed AKT1 phospho-variants into human cells. This system selectively induces the phosphorylation of its substrate (glycogen synthase kinase 3α) and downstream effector (ribosomal protein S6) at Ser9 and Ser240/244, respectively, in the PI3K/AKT/mTOR signaling pathway. Until now, traditional methods of phosphorylating and activating AKT via growth factors or insulin have resulted in the activation of multiple kinases, complicating the selective study of AKT1. These variants were generated by fusing AKT1 with an N-terminal cell penetrating peptide tag derived from the human immunodeficiency virus trans-activator of transcription (TAT) protein, and then expressed and purified from *E. coli*. The tag did not alter AKT1 kinase activity but facilitated the efficient and rapid delivery of AKT1 phospho-protein variants into human cells. These findings demonstrate an efficient delivery system for programmed AKT1 phospho-variants into human cells, thus providing a novel cell-based model system for specifically investigating AKT1 signaling activity.

Ghodsinia et al. (contribution 2) characterized two novel non-hotspot mutants at Q661K (exon 13) and C901R (exon 19) of the *PIK3CA* gene in NIH3T3 and HCT116 cells. This gene plays a crucial role in colorectal cancer (CRC) as it encodes the p110α catalytic subunit of PI3K, which is involved in cell growth and migration. Well-characterized mutations in hotspots of the *PIK3CA* gene, such as E545K (exon 9) and H1047R (exon 20), contribute differently to CRC progression. E545K promotes a wide range of oncogenic behaviors, while H1047R has a more limited scope, highlighting the importance of understanding the specific effects of each mutation within the gene. Their findings offer insights into the diversity of mutation effects within the same gene, affecting cellular functions such as proliferation, apoptosis resistance, and cytoskeletal reorganization, all crucial for cancer development and metastasis. These mutations may disrupt the interaction between the p110α catalytic subunit and the p85α regulatory subunit, leading to increased PI3K signaling and consequent cancer progression. Additionally, they influence RNA expression levels, tumor microenvironment, and the distribution of immune cells within the tumor. While many *PIK3CA* mutations in CRC occur in hotspot regions, they are not hereditary. The findings contribute to the growing body of knowledge that will help refine the prognostic and predictive value of *PIK3CA* mutations in CRC, with significant implications for improving patient outcomes. 

The activity of mTOR complex 1 (mTORC1) in the proliferative layer of healthy skin prevents differentiation of keratinocytes and is suppressed by the tuberous sclerosis complex (TSC) through its inhibition of the small GTPase, Ras homolog enriched in brain (Rheb), in other tissues [8]. However, in inflammatory conditions, cytokines disrupt this regulatory mechanism and prevent proper keratinocyte differentiation. Therefore, Ferreri et al. (contribution 3) investigated the role of proinflammatory cytokines in the regulation of TSC and the pathogenesis of psoriasis. Proinflammatory cytokines, such as tumor necrosis factor-α (TNF-α) and interleukin-1β (IL-1β), contribute to the pathogenesis of psoriasis. They found that TNF-α and IL-1β could induce phosphorylation of TSC2 at S939 via the PI3K/AKT and mitogen-activated protein kinase (MAPK) pathways in HaCaT keratinocytes. Surprisingly, phosphorylation of TSC2 S939 was not detected in the lesional psoriatic skin of the patients. Further in vitro studies showed that proinflammatory cytokines induce the dissociation of TSC2 from lysosomes, leading to its destabilization and degradation. This in turn results in chronic mTORC1 hyperactivation and impaired keratinocyte differentiation, thus contributing to the phenotypical changes seen in psoriatic epidermis. 

Overexpression or hyperactivation of the AKT pathway is a common event that contributes to the progression of breast cancer by promoting the survival and proliferation of breast cancer cells. However, little is known about the precise genome-wide transcriptomic changes associated with the AKT pathway in breast cancer cells. To clarify this, George et al. (contribution 4) conducted a genome-wide RNA-sequencing analysis after selectively knocking down AKT1 using specific siRNAs or inhibiting its activity with a pan-AKT inhibitor VIII in breast cancer cells. Alterations in AKT1 cellular levels impact the expression of a set of differentially expressed genes, which in turn affects its cellular functions. Surprisingly, AKT1 also plays an intrinsic role in suppressing the expression of a subset of genes in both unstimulated and growth factor-stimulated breast cancer cells. Consistently, the expression of this subset of genes also increases in breast tumors when AKT1 is depleted or not undetectable and decreases in breast tumors when AKT1 levels are elevated. Real-time polymerase chain reaction (qRT-PCR) analysis validated the RNA-sequencing data from two breast cancer cell lines and breast cancer patient-derived cells. The results provide insights into the AKT1-dependent modulation of gene expression in breast cancer cells and emphasize its importance in cellular function and potential therapeutic targets.

Wilczek et al. (contribution 5) studied the consequences of JCPyV infection on progressive multifocal leukoencephalopathy (PML). Using RNA sequencing and chemical inhibitors of PI3K, AKT, and mTOR, they showed that the PI3K/AKT/mTOR pathway is essential for JCPyV infection in primary astrocytes but not in transformed cell lines. The findings indicate that the pathway could be a potential target for developing therapeutics against PML and open possibilities for repurposing anticancer drugs that inhibit the PI3K/AKT/mTOR pathway for treatment of PML. 

Osteonecrosis of the femoral head (ONFH) induced by glucocorticoid use is a challenging condition to manage. Ma et al. (contribution 6) demonstrated a therapeutic potential of extracellular vesicles derived from bone marrow stem cells (BMSC-EVs) in the treatment of glucocorticoid-induced ONFH. They found that glucocorticoid inhibited autophagy by activating the PI3K/AKT/mTOR pathway, resulting in a decrease in cell viability and angiogenesis capacity and an increase in apoptosis of bone microvascular endothelial cells (BMECs). BMSC-EVs prevented glucocorticoid-induced injury of BMECs by suppressing the glucocorticoid-activated PI3K/AKT/mTOR pathway, promoting autophagy. 

The PDK1 signaling plays a crucial role in the radiotherapy resistance (IR resistance) observed in hepatocellular carcinoma (HCC), contributing to treatment failure. Bamodu et al. (contribution 7) suggest that PDK1 is pivotal in promoting IR resistance by enhancing DNA damage repair and facilitating post-IR relapse in aggressive HCC cells. The aberrant expression of PDK1 in poorly differentiated HCC cells, in contrast to well-differentiated or normal liver cells, underscores its role in activating the PI3K/AKT/mTOR signaling pathway, which is significant. This activation enables cells to evade IR toxicity, leading to enhanced survival, proliferation, and a dedifferentiated phenotype that is highly resistant to radiotherapy. Molecular ablation of PDK1 function increased the susceptibility of HCC cells to IR and suppressed PI3K/AKT/mTOR signaling. Therefore, targeting PDK1 could potentially enhance radiosensitivity in HCC treatment. The positive correlation between PDK1-driven IR resistance and factors such as cell motility, invasiveness, and stemness marker expression further supports its critical role in this resistance development. Upregulation of stemness markers like ALDH1A1, PROM1, SOX2, KLF4, and POU5F1, increased tumor sphere-formation efficiency, and suppressed biomarkers of DNA damage suggest a complex network of signaling, contributing to the radioresistant phenotype. This preclinical evidence implicates PDK1 as an active driver of IR resistance through the activation of PI3K/AKT/mTOR signaling pathway, modulation of cancer stemness signaling, and suppression of DNA damage. This underscores the potential of PDK1-targeted therapies to enhance radiosensitivity in HCC treatment. These findings provide valuable insights into the molecular mechanisms underlying IR resistance in HCC and open new avenues for therapeutic intervention. It will be intriguing to see translational research in clinical settings to determine whether PDK1-targeted therapies can indeed improve outcomes for patients with IR-resistant HCC. 

Inhibition of Notch signaling through γ-secretase inhibitor (GSI) treatment activates the AKT/mTOR signaling pathways, critical for survival, differentiation, myogenesis, and muscle protein synthesis (MPS) in myotubes. Huot et al. (contribution 8) explored the potential dependency of GSI’s impact on myogenesis and MPS via the AKT/mTOR signaling in C2C12 cells by assessing myotube formation, anabolic signaling, and MPS after exposing C2C12 cells to GSI along with rapamycin and API-1, inhibitors of mTOR and AKT, respectively. Rapamycin and API-1 counteracted GSI-mediated effects on myotube formation and fusion in C2C12 cells, whereas GSI treatment rescued MPS and GSK3β Ser9 phosphorylation in C2C12 cells treated with rapamycin and API-1. These findings suggest that GSI treatment rescues MPS in C2C12 myotubes independently of AKT/mTOR signaling, possibly by modulating the phosphorylation of GSK3β. 

Luo et al. (contribution 9) investigated the mechanism of anticancer effects of dihydroartemisinin (DHA), an anti-malarial drug, using rhabdomyosarcoma (RMS) cells as an experimental model. They found that DHA inhibits the mTORC1 signaling pathway in RMS cells but not in normal cells. Mechanistically, DHA does not directly bind to mTOR or FKBP12, nor does it inhibit IGFR, PI3K, and ERK1/2 pathways, or activate phosphatase and tensin homolog (PTEN). Instead, DHA activates AMP-activated protein kinase (AMPK). Inhibition of AMPK, either pharmacologically or genetically, attenuates DHA’s inhibitory effect on mTORC1. Also, DHA causes the dissociation of raptor from mTOR, crucial for inhibiting mTORC1 activity. Oral treatment with artesunate, a prodrug of DHA, effectively suppresses the tumor growth of RMS xenografts by activating AMPK and inhibiting mTORC1. These findings support the idea that DHA has a great potential for treatment of RMS. The study underscores the importance of understanding the molecular mechanisms of drugs, enabling their repositioning for treatments beyond their original indications.

mTORC2 is essential for cellular growth and metabolism through the phosphorylation of AGC family kinases such as AKT and protein kinase C (PKC) at their hydrophobic motif (HM), which is crucial for their activation. The mitogen activated protein kinase/extracellular signal-regulated kinase (MAPK/ERK) pathway also significantly contributes to cell proliferation and differentiation by phosphorylating p90 ribosomal S6 kinase (RSK) at Ser380, a conserved HM site within the AGC kinase family. The study by Chou et al. (contribution 10) demonstrates the complexity of these interactions by showing that RSK phosphorylation at Ser380 can occur independently of mTOR’s catalytic activity, although optimal phosphorylation of RSK at this site requires an intact mTORC2. This finding suggests that mTORC2 may primarily serve as a scaffold to facilitate this process. This phosphorylation event at Ser380 is critical as it potentially influences the substrate specificity of RSK and indicates how RSK can respond to alterations in nutrient availability. This example highlights the fascinating ability of cells to integrate multiple signals to maintain equilibrium and adapt to environmental changes. 

Hsueh et al. (contribution 11) elucidated the protective effects of ascorbic acid (AA) against oxidative stress in corneal endothelial cells. Using in vitro and in vivo models, they demonstrated the mechanisms by which AA mitigates damage from paraquat-induced reactive oxygen species (ROS) and improves corneal health. The downregulation of AKT phosphorylation in response to oxidative stress and its attenuation through AA pretreatment is a significant finding, highlighting the potential role of the PI3K/AKT pathway in protecting corneal endothelium. The in vivo rabbit corneal damage model further supports the topical application of AA as a viable perioperative strategy to enhance corneal clarity and prevent oxidative damage. These findings provide a valuable contribution to ophthalmology, especially for patients undergoing procedures like phacoemulsification who are at risk of oxidative stress-induced corneal endothelial decompensation. This represents a promising step towards improving surgical outcomes and patient vision post-surgery. 

Galectin-1 (GAL1) is a β-galactoside-binding protein involved in multiple aspects of tumorigenesis. Su et al. (contribution 12) highlight the significant role of GAL1 in upper tract urothelial carcinoma (UTUC). GAL1 expression is associated with worse outcomes in UTUC, indicated by poorer recurrence-free survival (RFS) and cancer-specific survival (CSS) in patients with higher GAL1 levels. In vitro studies conducted on four urothelial carcinoma (UC) cell lines (BFTC-909, T24, RT4, and J82) suggest that GAL1 promotes tumor invasiveness and migration, potentially through its effects on proteins like MMP-2, MMP-9, and TIMP-1, which are involved in the breakdown and remodeling of the extracellular matrix—a key process in cancer metastasis. Overexpression of GAL1 activates the FAK/PI3K/AKT/mTOR pathway, which correlates with disease progression and patients’ survival in upper urinary urothelial carcinoma. These suggest GAL1 as a potential therapeutic target, given that its modulation could affect crucial pathways involved in cancer progression. Moreover, leveraging public genomic data from TCGA and GSE32894 for comparison adds a valuable dimension, broadening the understanding of GAL1’s role in UTUC. It is evident that GAL1’s function in cancer biology is complex and multifaceted, influencing various aspects of tumor growth and metastasis. Hence, this study contributes significantly to the growing body of evidence highlighting GAL1’s importance in cancer and may lead to novel therapeutic strategies in UTUC. 

The treatment and recovery from myocardial infarction (MI) are significantly influenced by the PI3K/AKT signaling pathway. Activation of this pathway is crucial as it provides cardioprotection, enhances cell survival, and mitigates the negative consequences of post-infarction myocardial remodeling. Walkowski et al. (contribution 13) reviewed the role of the PI3K/AKT pathway in each step of ischemia and subsequent left ventricular remodeling. They described a notable impairment in the function of the PI3K/AKT pathway in diabetes, which exacerbates adverse changes in the myocardium following an infarct. This impairment is a notable concern, given the frequent coexistence of diabetes and cardiovascular diseases in patients, potentially compromising the efficacy of treatments targeting the PI3K/AKT pathway. They also discuss some cardiac and antidiabetic drugs, which can activate or inhibit different components of the PI3K/AKT pathway, influencing myocardial ischemia and left ventricular remodeling. It is important to note that while there are promising substances and drugs that interact with the PI3K/AKT pathway, more investigation is needed before they can be introduced into clinical practice. This includes understanding the molecular mechanisms of their effects and conducting extensive clinical trials to ensure safety and efficacy for patients with MI, especially those with diabetes. The interplay between diabetes, cardiac conditions, and the PI3K/AKT pathway is complex, and personalized medicine approaches may be required to optimize treatment strategies for individual patients. 

In a review article, Loissell-Baltazar and Dokudovskaya (contribution 14) gave a summary of the key points regarding the Seh 1 associated (SEA) complex. The SEA complex in yeast and its homologue in humans (the GATOR complex) are crucial upstream regulators of mTORC1 that integrate various signals from amino acids, growth factor, oxygen, and DNA-damaging agents to regulate cellular responses to stress. The complex plays a pivotal role in nutrient sensing and response, influencing the cell’s decision to pursue metabolic pathways, which are critical for cell growth, metabolism, and autophagy. Mutations in this complex disrupt the normal functioning of the mTORC1 pathway, leading to pathological conditions. The ongoing research in the SEA/GATOR complex not only enhances our comprehension of cellular metabolism and growth but also underscores the complex’s potential as a therapeutic target for related diseases. It is a prime example of how fundamental research can lead to breakthroughs in understanding and potentially treating complex diseases.

Transcription factor EB (TFEB) is a master regulator of autophagy and lysosomal biogenesis; thus, its ability to clear intracellular pathogens makes it a suitable therapeutic target for pathologies related to autophagy dysfunction. The identification of TFEB agonists and their progression into preclinical and clinical studies mark significant advancements in this area. In a review article, Chen et al. (contribution 15) discussed TFEB regarding its structure, regulatory mechanisms, and implications in diseases, as well as a variety of TFEB agonists. This review underscores the importance of TFEB in managing diseases associated with autophagy dysregulation and suggests that modulating TFEB activity could be a promising therapeutic approach. As research continues, we can expect to see further developments in the use of TFEB agonists for the management of human diseases.

## 3. Conclusions

Overall, the 15 papers published in this Special Issue highlight the importance of the PI3K/AKT/mTOR pathway in human health and diseases. In summary, a comprehensive understanding of the physiological and pathophysiological functions of this pathway is of great importance for the development of effective strategies to better manage human health and diseases.
